# Scientific Items

**Published:** 1877-01-20

**Authors:** 


### Scientific Items.

*Sideraphite* is a new alloy composed of iron, tungsten, nickle, copper and aluminum. The vegetable acids do not affect it, and the mineral acids very slightly. It is cheap, yet more useful than genuine silver.

*The Alpha fibre* plant is said to be very largely cultivated in the French colony of Algeria, millions of acres being devoted to this purpose. It is used for the manufacture of paper, for which it is better and cheaper than rags.

*Among* the papers of Professor O. C. Marsh, in regard to fossils in the museum of Yale College, is a description of the gigantic flying lizards, the remains of which have been found in Kansas. He says that the larger of them had a spread of wings of not less than twenty-five feet. They were found in the chalk formation, and hence existed at a very remote period of time.

*Notwithstanding* the failure of the recent Arctic expedition, Dr. Peterman thinks that a navigable route to the Pole will yet be discovered. He is of the opinion that Greenland extends northward to the very pole itself.

